# Necrotizing fasciitis resembled acute coronary syndrome: A case report

**DOI:** 10.1002/ccr3.9513

**Published:** 2024-10-31

**Authors:** Matin Sepehrinia, Faeze Yousefi, Adib Valibeygi, Abdulhakim Alkamel

**Affiliations:** ^1^ Student Research Committee Fasa University of Medical Sciences Fasa Iran; ^2^ Non‐Communicable Diseases Research Center Fasa University of Medical Sciences Fasa Iran; ^3^ Department of Cardiovascular Disease, Faculty of Medicine Fasa University of Medical Sciences Fasa Iran

**Keywords:** cardiogenic shock, case report, myocardial infarction, necrotizing fasciitis, septic shock

## Abstract

Chest pain is a frequent complaint in emergency departments, with various differential diagnoses from benign to life‐threatening. Hereby, we described a 60‐year‐old man presented with chest pain and hypotension who initially misdiagnosed as acute coronary syndrome, but was ultimately diagnosed with necrotizing fasciitis. This case highlights the importance of considering rare causes of chest pain.

## 1 INTRODUCTION

Necrotizing fasciitis (NF) is a progressive and frequently fatal soft tissue infection involving the skin, fascia, and muscles. The estimated prevalence of this rare disease ranges from 0.4 to 15.5 cases per 100,000 individuals, with a mortality rate of approximately 21.5%.^1^, ^2^ Trauma is the primary cause of NF, as it compromises the skin's protective barrier and allows pathogens to enter the tissue.^3^ NF is more common among men and individuals with diabetes mellitus, often occurring during the summer months.^4^, ^5^ Nearly 70% of NF cases involve the lower extremities. The main pathogens responsible for NF are staphylococci, streptococci, and other gram‐negative bacteria. Early symptoms of NF include redness, swelling, and localized pain, progressing to tenderness, bullae, vesicles, and tissue necrosis. Patients may also present systemic symptoms such as tachycardia, tachypnea, fever, and hypotension. Given the aggressive nature of NF, timely diagnosis and immediate initiation of antimicrobial therapy and surgical debridement are essential for successful treatment.^2^, ^3^, ^6^

Chest pain is a common reason for emergency department visits and can pose a diagnostic challenge for physicians due to the wide range of potential causes, ranging from benign conditions to life‐threatening emergencies like acute coronary syndrome (ACS), pulmonary embolism, aortic dissection, and esophageal rupture.^7^ Herein, we describe a case of a previously healthy 60‐year‐old man who presented with chest pain and hypotension, ultimately diagnosed with NF.

## 2 CASE HISTORY/EXAMINATION

A 60‐year‐old man without any prior medical history presented to a small‐town emergency department complaining of chest pain that started 4 h prior to admission. The patient described the chest pain as severe, continuous, compressive, and left‐sided, radiating to the left hand, aggravated by physical activity, and relieved by taking nitroglycerine. The pain was also accompanied by diaphoresis and nausea. The patient denied any history of fever, dyspnea, palpitation, and chest trauma. He did not have any history of prior hospital admission, surgery, diagnosed medical disease, or medication use. The patient quit smoking cigarettes 10 years ago and stopped using opium 3 months ago. Upon admission, he was reported to be in a shock state although resuscitation and intravenous norepinephrine infusion had been initiated. Given his condition, cardiogenic shock resulting from ACS was suspected. The patient was therefore instantly administered with aspirin and subsequently transferred to a university‐based hospital.

On arrival, his blood pressure was 85/55 mmHg while receiving norepinephrine, his pulse rate was 103 beats per minute, and the temperature was 36.9°C. Physical examination revealed no signs of deformity, erythema, or trauma on the chest. The lower extremities were cold. Abdominal examination and heart and lung auscultation were unremarkable.

### 2.1 Differential diagnosis, investigations, and treatment

Considering the chest pain and unstable hemodynamics despite using a vasopressor, the differential diagnoses included cardiogenic shock due to acute myocardial infarction or myocarditis, pulmonary embolism, aortic dissection, and pericarditis complicated with tamponade.

**FIGURE 1:**
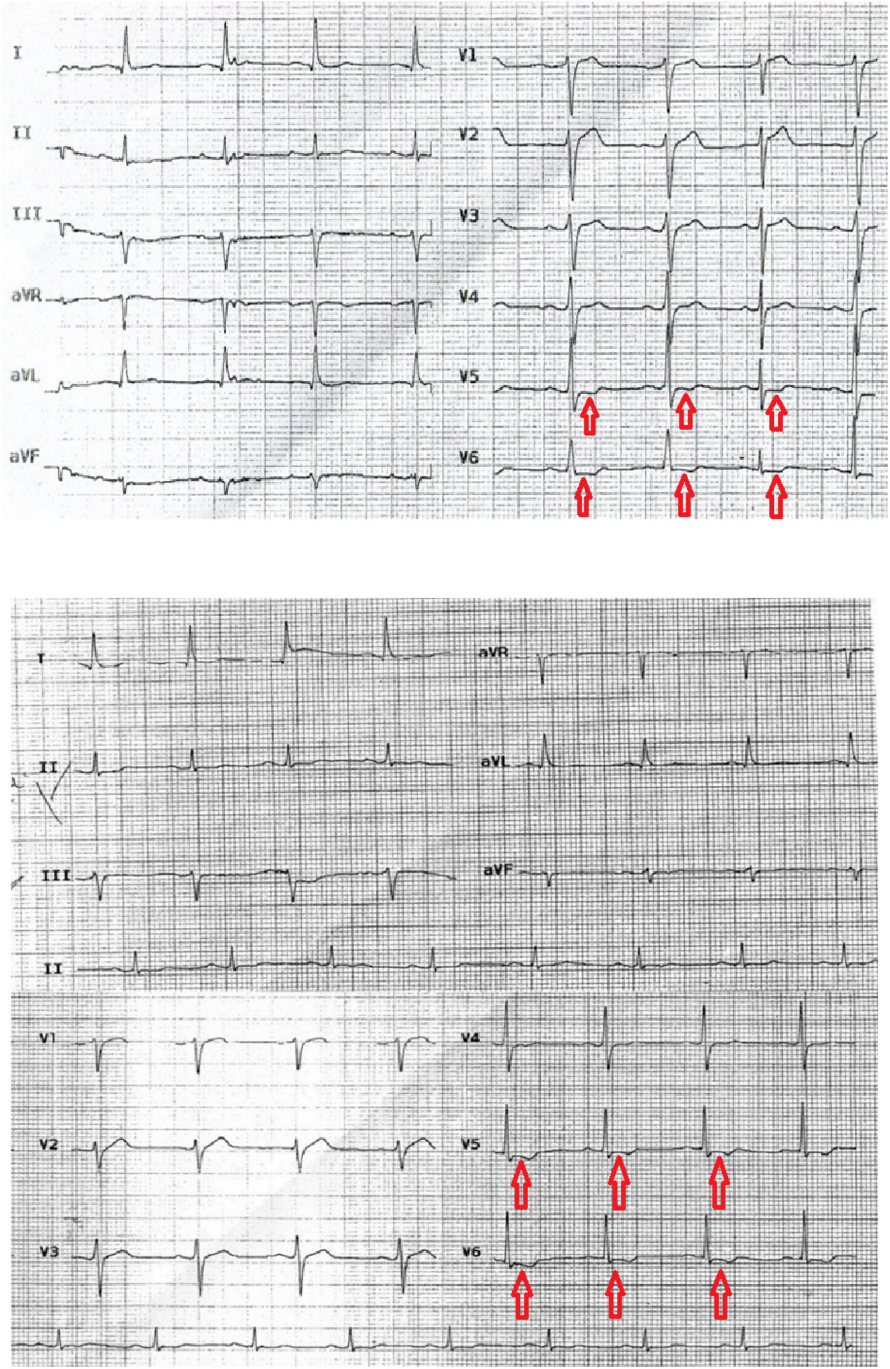
The first ECG showed ST‐depression in V5 and V6. The second ECG showed T inversion and ST‐depression in V5 and V6.

The first electrocardiogram (ECG) showed ST depression in V5 and V6 (Figure 1). The next ECG, before transferring, showed T inversion in V5 and V6, but the ST depression resolved. Trans‐thoracic Echocardiography (TTE) demonstrated a normal‐sized left ventricle with severe systolic dysfunction (left ventricular ejection fraction (LVEF): 15%–20%) and global hypokinesia. Also, an enlarged right ventricle with mild dysfunction, mild mitral regurgitation, and mild to moderate tricuspid regurgitation were observed.

**FIGURE 2:**
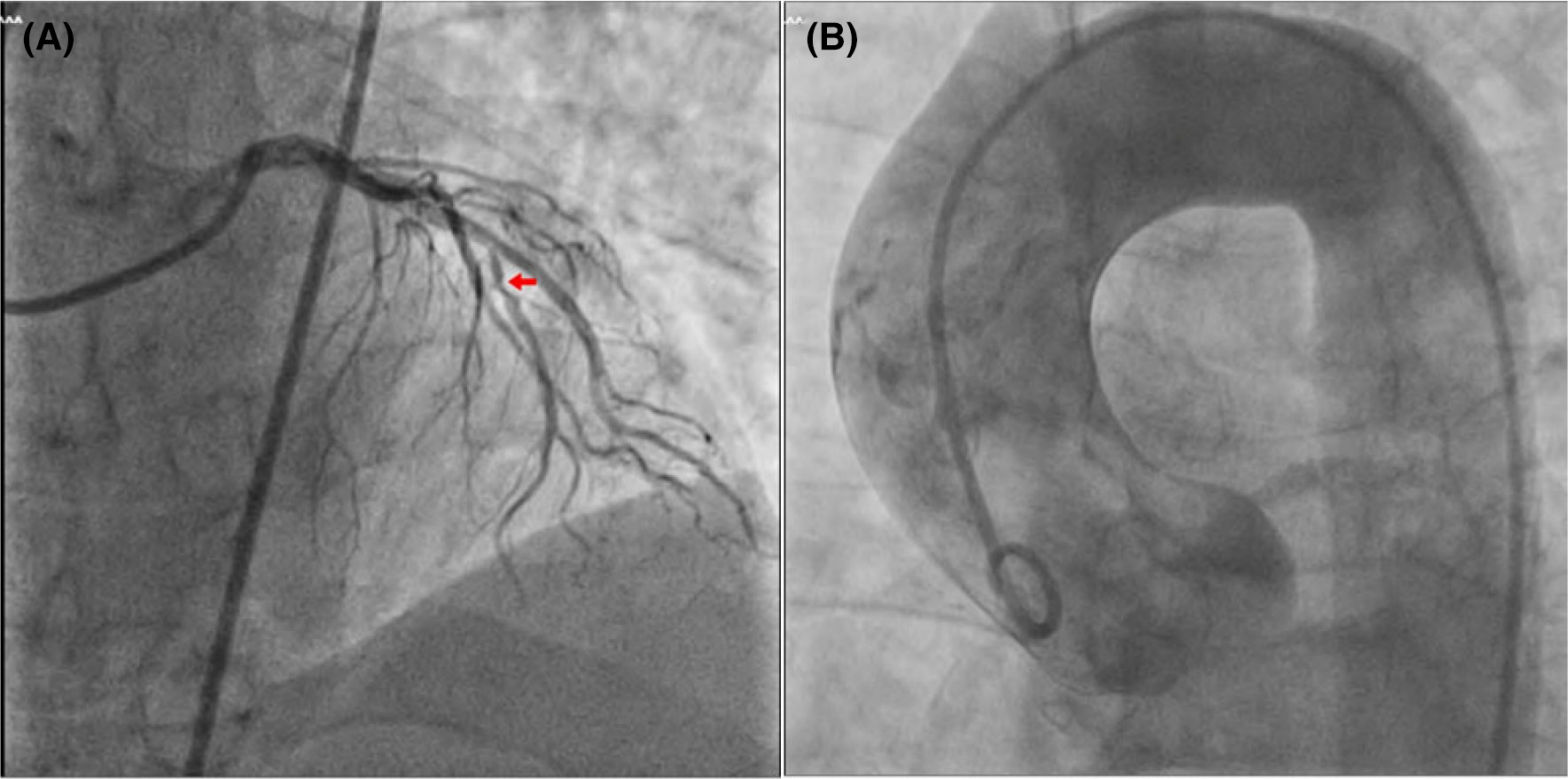
Coronary angiography and aortography were performed for the patient. Coronary angiography revealed significant stenosis in the left anterior descending artery (red arrow) (A). On the other hand, aortography was found to be normal and it failed to find any sign of aortic dissection (B).

Severe and persistent chest pain mimicking ACS, unresponsive to opioid analgesics, along with unstable hemodynamics, and a sudden drop in LVEF, convinced the cardiology team to perform a coronary angiography urgently. The procedure revealed significant stenosis in the mid‐part of the left anterior descending artery (Figure 2A). Simultaneously, an aortography was done in the catheterization laboratory which showed no evidence of dissection (Figure 2B). Non‐culprit revascularization of the left anterior descending artery was carried out to alleviate chest pain and improve hemodynamics.

Laboratory results were remarkable for a troponin level of 1.5 ng/dL (normal range: <2 ng/dL), a D‐dimer level of 280 ng/mL, serum creatinine of 2.4 mg/dL, a blood urine nitrogen of 68 mg/dL, a C‐reactive protein level of 56 mg/dL, and the white blood cell count of 10.4 10^3^/μl (neutrophil: 92.7%).

After ruling out the aforementioned differential diagnoses based on the ECG, TTE, coronary angiography, and laboratory tests, suspicion of septic shock arose due to elevated C‐reactive protein level, shock state, acute kidney injury, and acute heart failure. Empiric antibiotic therapy (linezolid, ciprofloxacin, clindamycin) was initiated in consultation with an infectious disease specialist. Also, further paraclinical investigations including a computed tomography scan of the lungs, blood cultures, liver enzymes, urine analysis, and urine culture failed to identify the source of the infection.

**FIGURE 3:**
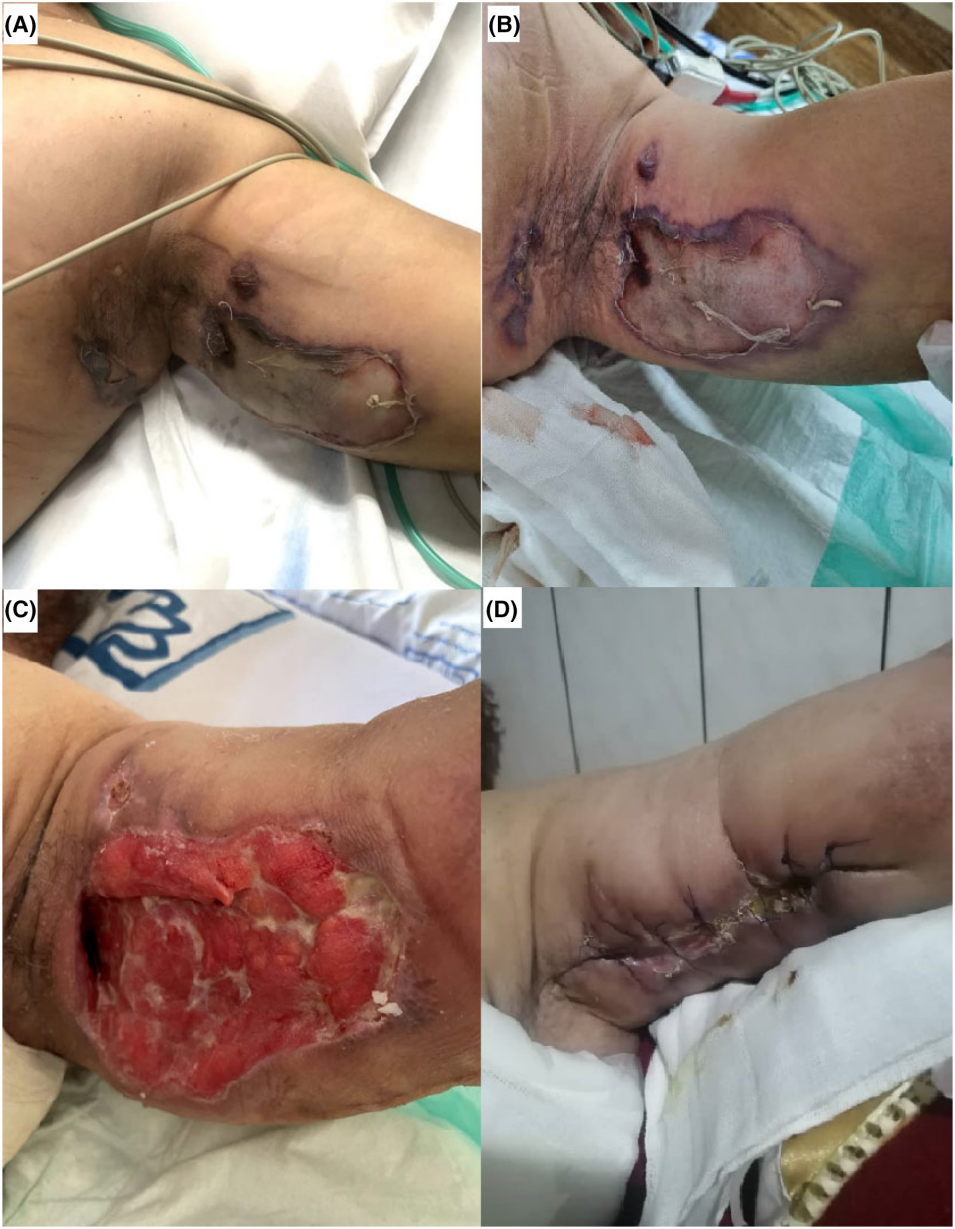
These figures depict the patient's wound in his left axilla after the bullae rupture (A and B), after the first debridment (C), and 1 week after discharging from hospital (D).

**FIGURE 4:**
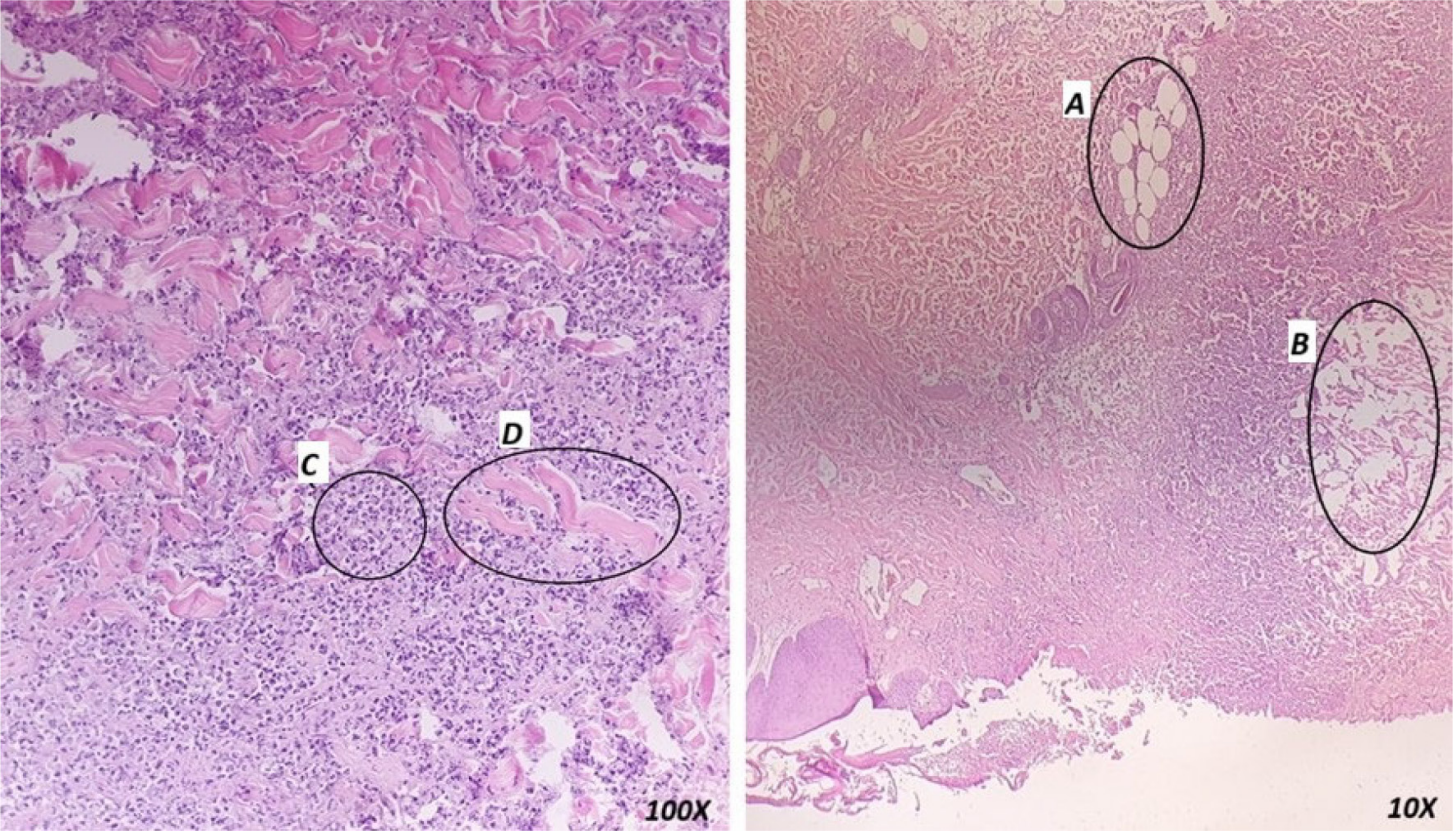
The pathological examination showed necrotic fascial tissue with inflamed adipose tissue and micro‐abscess formation at dermis subcutis interface. The overlying skin is moderately inflamed and edematous. These findings are consistent with clinical impression of necrotizing fasciitis. (A) intact septated adipocytes, (B) necrosis of subcutaneous fat; (C) infiltrated neutrophils, (D) degenerated wavy form muscle fibers surrounded with neutrophils (resolution: 144 dpi).

On the second day of admission, the patient developed a fever with a bullae that ruptured with necrotic borders in the left axilla (Figure 3A,B). Also, below the wound, the skin got tense and erythematous extending toward the flank. The soft tissue sonography revealed fat inflammation. Suspecting the diagnosis of NF, the patient underwent biopsy, surgical debridement of the necrotic and infectious foci, and antibiotic therapy with piperacillin/tazobactam + clindamycin + linezolid. The white blood cell count and C‐reactive protein level rose to 23.5 10^3^/μl and 75.2 mg/dL, respectively. Meanwhile, the patient experienced transient hepatic dysfunction. ALT rose from 24 mg/dL to 201 mg/dL and then decreased to 78 mg/dL. International normalized ratio (INR) rose from 1.08 (prothrombin time (PT): 13.5 s) to 1.47 (PT: 18.38 s) and then decreased to 1.2 (PT: 15 s). Afterwards, the hemodynamic status improved gradually and he was weaned from vasopressor. The patient underwent two more surgical debridements to remove the necrotic foci (Figure 3C). The wound biopsy confirmed the diagnosis of NF (Figure 4) and the patient was continued on the antibiotic therapy.

### 2.2 Outcome and follow‐up

After 32 days of hospitalization, echocardiography revealed an improved cardiac systolic function (LVEF = 55%). Finally, the patient was discharged in a stable condition after 37 days of hospitalization and demonstrated complete wound healing at an eight‐month follow‐up (Figure 3D shows the wound healing 1 week after discharging from hospital).

## 3 DISCUSSION

The described case exhibited an atypical presentation of NF and shared common features with other life‐threatening causes of chest pain, which complicated the diagnosis. The unusual location of NF, lack of predisposing factors such as diabetes, absence of prior trauma or surgery, lack of fever, and chest pain mimicking ACS all contributed to diagnostic challenges. The absence of fever could be attributed to the use of non‐steroidal anti‐inflammatory drugs (NSAIDs) or acetaminophen for pain management before admission. Additionally, NF caused by clostridium sordellii may not always present with fever. Additionally, in cases where spontaneous NF originates in deep fascia, cutaneous signs may not be evident in the early stages. Furthermore, systemic manifestations of NF may be mistakenly attributed to other causes.^2^, ^3^ Another challenge in diagnosing and treatment of this case was negative blood cultures. Surprisingly, most cases of NF have negative blood cultures.^1^, ^8^, ^9^ This might be due to unusual pathogens or antibiotic consumption prior to culture.^10^

This case was not the only instance of a patient with NF presenting with chest pain and being misdiagnosed as cardiac disease. A similar case involved a 75‐year‐old woman who presented with chest pain in the emergency department, initially suspected to be myocardial infarction, but further investigation revealed NF in the chest wall.^11^ Additionally, in another case report, a 59‐year‐old man complained of chest pain following blunt trauma. On physical examination, the patient exhibited tachycardia, hypotension, and a temperature of 37.5°C, with tenderness and erythema on the chest wall. Laboratory findings showed leukocytosis with bandemia, elevated liver enzymes, serum creatinine, and troponin levels. The ECG displayed ST elevation in multiple leads, including I, II, aVL, and V2 to V6. Echocardiography indicated an LVEF of 40% with global hypokinesia. Coronary angiography revealed no significant stenosis in the coronary arteries. Ultimately, the patient was diagnosed with sepsis due to NF in the chest wall.^12^

In our case, NF caused multi‐organ damage due to the septic shock based on the “Sepsis‐3” definition.^13^ Cardiovascular dysfunction typically presents as hypotension due to hypovolemia, decreased cardiac contractility, and vascular tone. Myocardial depression manifests as increased end‐diastolic volume index and reduced ejection fraction, both of which are reversible.^14^, ^15^ Renal dysfunction presents as oliguria, increased creatinine, and blood urea nitrogen due to hypo‐perfusion or sepsis. Liver dysfunction presents as increased bilirubin or aminotransferase enzymes due to cytokines and toxins released by pathogens. The underlying mechanisms of organ failure in sepsis include circulatory abnormality, endothelial dysfunction, inappropriate host immune response, abnormal cellular oxygen metabolism, mitochondrial dysfunction, and activation of cell death pathways like necrosis and apoptosis.^15^

One of the challenging decisions in managing this patient was determining whether he needed angiography. The patient was in a state of shock with severe chest pain and the ECG showed transient ST depression and T inversion. Transient ST change or T inversion could be indicative of ACS and should be managed with early coronary angiography.^16^ Additionally, an early invasive strategy should be considered for refractory chest pain and hemodynamic instability in patients with unstable angina.^17^

### 3.1 Limitations

The unavailability of short‐term mechanical circulatory support at our facility restricted our capacity to enhance cardiac output and improve end‐organ perfusion.

## 4 CONCLUSION

Our patient presented with NF, manifesting as chest pain and hypotension, leading to a misdiagnosis of cardiac diseases. This case report highlights the significance of considering rare differential diagnoses and promptly investigating to rule out life‐threatening possibilities, ultimately reaching an accurate diagnosis.

## AUTHOR CONTRIBUTIONS

**Matin Sepehrinia:** Project administration; writing – original draft. **Faeze Yousefi:** Resources; writing – original draft. **Adib Valibeygi:** Writing – original draft. **Abdulhakim Alkamel:** Supervision; writing – review and editing.

## FUNDING INFORMATION

No financial disclosures to make.

## CONFLICT OF INTEREST STATEMENT

The authors declare that they have no conflict of interest.

## CONSENT

A written informed consent has been obtained from the involved patient. The patient has given approval for this information to be published in this case report. Also, this case report was approved by the ethics committee of Fasa University of Medical Sciences (FUMS) (Approval Code: IR.FUMS.REC.1402.097).

## Data Availability

Data sharing not applicable to this article as no datasets were generated or analysed during the current study.
